# The Prevention of Bio-Organic Fertilizer Fermented from Cow Manure Compost by *Bacillus* sp. XG-1 on Watermelon Continuous Cropping Barrier

**DOI:** 10.3390/ijerph17165714

**Published:** 2020-08-07

**Authors:** Hao Zhang, Zi-Wei Hua, Wen-Zhi Liang, Qiu-Hong Niu, Xiang Wang

**Affiliations:** 1 School of Life Science and Technology, Nanyang Normal University, Nanyang 473061, China; zhanghao660@nynu.edu.cn (H.Z.); 15239943441@139.com (Z.-W.H.); qiuhongniu723@163.com (Q.-H.N.); 2 Fengyang Fengshuo Agricultural Development Co., Ltd., Chuzhou 233100, Anhui, China; 17656655327@139.com; 3 College of Resource and Environment, Anhui Science and Technology University, Chuzhou 233100, Anhui, China

**Keywords:** *Bacillus* sp. XG-1, antagonists, growth promotion, bio-organic fertilizer, continuous cropping barrier

## Abstract

The continuous cropping barrier is an important factor leading to the decline of watermelon quality and yield. In this study, we focused on a bio-organic fertilizer prepared with one bacterial strain, *Bacillus* sp. XG-1, to prevent the occurrence of the continuous cropping barrier. The strain XG-1 was isolated from watermelon rhizosphere soil, and promoted the growth of watermelon by producing phytase (0.19 U/mL), indole-3-acetic acid (IAA, 7.31 mg/L), and gibberellins (GA3, 2.47 mg/L). In addition, the strain also possessed a strong antagonistic effect against the pathogen *Fusarium oxysporum* f. sp. *niveum* (Fon) by inhibiting conidia germination with an inhibition ratio of 85.3% and mycelium growth. The bio-organic fertilizer fermented by XG-1, based on cow manure compost and rapeseed meal (85:15, w/w) under optimal conditions, was mixed in soil (watermelon had been planted for two consecutive years). After the cultivation of watermelon for 50 d, a higher density of XG-1 (9.79 × 10^5^ colony-forming units (CFU)/g) and one order of magnitude lower of Fon (1.29 × 10^3^ copies/g) were detected in the rhizosphere soil compared with soils without bio-organic fertilizer (7.59 × 10^4^ copies/g for Fon), leading to an 86.4% control efficiency of watermelon caused by *Fusarium* wilt. The application of bio-organic fertilizer enriched soil nutrients, including the organic matter (13.2%), total nitrogen (13.9%), total phosphorus (20.5%), and total potassium (3.77%), adjusted the soil pH from 6.69 to 7.01, and significantly improved the watermelon growth in terms of the seedling height, root length, fresh weight of seedling and root with increase of 78.8%, 72.2%, 84.6%, and 96.4%, respectively. This study regarded the watermelon continuous cropping soil as the research point, and focused on inhibiting Fon, regulating soil properties and enhancing watermelon growth to eliminate the continuous cropping barrier through a combination of compost and functional strains, demonstrating the potential application value in watermelon production.

## 1. Introduction

Watermelon (*Citrullus lanatus* Thumb.) is one of the most popular fruits in summer, and China is the largest watermelon-producing area in the world [1]. Under the premise of the current food strategy, how to use every part of cultivated land rationally and improve the land availability is a major issue. Improving the land availability will inevitably lead to the continuous cropping barrier, especially for watermelon [2]. The continuous cropping barrier refers to the phenomenon of plant growth and yield decrease, quality deterioration, soil-borne diseases and insect pests associated with the continuous cultivation or planting of the same crops, which is also called replant disease or continuous cropping disease. To realize barrier-free planting is one of the most difficult problems in the watermelon industry. The reasons for the watermelon continuous cropping barrier are complex, and they are the result of the comprehensive effect of many factors within the watermelon and soil systems: (1) the accumulation of self-toxic substances, such as benzoic acid and cinnamic acid, has a toxic effect on the watermelon root growth, and acidifies the soil pH [3]. (2) The extensive use of pesticides and chemical fertilizers, coupled with improper cultivation measures, leads to the accumulation of salt on the soil surface. In addition, watermelon roots distributed to the same range and depth, absorbed the same category of nutrients, resulting in a lack of specific nutrients in the soil [4]. (3) Watermelon *Fusarium* wilt caused by Fon wreaks havoc at all growth stages causing plants to wilt, followed by death, and it is difficult to control due to its strong survival ability [5]. At present, there are no effective control methods. Traditional measures, such as the cultivation of resistant plants and rotation, have no clear effects. Soil disinfection will cause pollution to the environment, and stimulate the formation of resistance in indigenous microorganisms [6].

Biological control is considered to be a stable, efficient, green and environmental protection means. Many microorganisms have been proved to control soil borne diseases, such as *Bacillus* sp. [7,8,9], *Paenibacillus* sp. [10], *Pseudomonas* sp. [11], and *Streptomyces* sp. [12]. Among antagonistic strains, the species of *Bacillus* possess significant advantages, including rapid growth, strong viability, a broad antibacterial spectrum, root colonization, etc., which can provide high-quality strain resources for agricultural production and improving the soil environment [13]. There also exist a series of reports on the inhibition against Fon by the species of *Bacillus*. Treated with *Bacillus subtilis* XG-1, Fon showed obvious mycelial deformation and 72.9% of its conidia germination was strongly inhibited [14]. *Bacillus* sp. WB could inhibit the growth of Fon by affecting the cellular morphology and disrupting the cell walls and membranes. The inhibition rate of WB against Fon was 82% [15]. The cell-free supernatant of *Bacillus amyloliquefaciens* LZN01 showed antagonistic behavior against Fon, leading to cellular morphological changes, including concave formations on the conidial surface, the disruption of cell walls and membranes, the leakage of intracellular contents and the aggregation of organelles [16].

The preferred forms for adding functional microorganisms to farmland soil are liquid and solid microbial agents. Considering that the antagonistic strains, especially the genus of *Bacillus*, are easier to agglomerate and self-disperse in the liquid medium, this is clearly not suitable for agricultural production. Therefore, producing bio-organic fertilizer through solid fermentation is regarded as a practical method for controlling the watermelon continuous cropping barrier or other soil borne diseases. The bio-organic fertilizer based on a mixture of amino acid fertilizer and pig manure composts (2:3, w/w) fermented by *Bacillus amyloliquefaciens* W19 could significantly decrease the *Fusarium* wilt disease of bananas [7]. Bio-organic fertilizer JDF35 (BOF) was generated by inoculating *Bacillus amyloliquefaciens* JDF35 into the organic fertilizer, and the *Fusarium* wilt incidence of watermelon in the BOF treatment was lower than that in the other treatments [17]. The novel bio-organic fertilizer, using Fen-liquor Daqu (FLD) as a fermentation starter, favorably altered the composition of the watermelon rhizosphere microbial community, suppressing *Fusarium* wilt disease and promoting quality [18].

At present, the solid-state fermentation substrate mainly contains chicken manure, pig manure, and rabbit manure [19,20,21]. However, with the increase of people’s demand for milk and beef products, the total number of cows reached 1.5 billion in 2017 across the world, which inevitably generated a large amount of cow manure, regarded as a potential source of fertilizer due to a variety of nutrient elements [22]. Because of poor air permeability, it is difficult and inefficient to decompose the organic matter in cow manure with traditional landfill methods. In the existing treatment methods, composting is considered to be an effective way to transform the waste into available fertilizer by microorganisms depending on the enzymes, and could also give specific functions of organic manure, such as antagonism. However, there exists little research on the fermentation of cow manure compost to produce bio-organic fertilizer, and it is mainly the mixture of cow and chicken manure that serves as a solid fermentation substrate [17,18]. 

Previous studies emphasized the control of Fon, the pathogen of watermelon wilt [23,24,25], but many inducements caused continuous cropping barrier, and the single control has limited effects on prevention. Therefore, it is necessary to find a lasting and comprehensive control method. In view of the advantages of *Bacillus* in agricultural biological control and the current situation of cow manure accumulation, in this study, we focused on the isolation of the species of *Bacillus* from watermelon rhizosphere soil, identification of its growth promoting substances and antagonistic effects. The novel bio-organic fertilizer prepared by the second fermentation of cow manure compost using strain XG-1 was added to watermelon continuous cropping soil, expounding the application potential for the prevention of the continuous cropping barrier from the growth of watermelon, the improvement of soil properties, and biocontrol of Fon.

## 2. Materials and Methods

### 2.1. Media, Chemicals, Compost and Soil

The Luria-Bertani medium (LB, g/L), used for *Bacillus* strain cultivation consisted of yeast extracts 5.0, tryptone 10.0, and NaCl 10.0. The Minimal Salt Medium (MSM, g/L) for the investigation of the effect of root exudates on *Bacillus* strain growth, contained NH_4_NO_3_ 1.5, KH_2_PO_4_ 0.5, K_2_HPO_4_·3H_2_O 1.5, NaCl 1.0, and MgSO_4_ 0.2. The Landy medium was used for indole-3-acetic acid (IAA) production [26]. The Nutrient broth medium (NB) and Potato Dextrose Broth medium (PDB), purchased from Shanghai Bio-way technology Co., Ltd., Shanghai, China, were used for gibberellic acid (GA3) and Fon conidial production. Semisolid chemotactic buffer for chemotaxis experiments contains phosphate buffer saline (PBS, 100 mM pH 7.0), EDTA (20 µM) and agar (0.4%, w/w). Hoagland medium (g/L), used for watermelon growth, contained Ca(NO_3_)_2_ 0.90, KNO_3_ 0.51, NH_4_NO_3_ 0.08, KH_2_PO_4_ 0.14, MgSO_4_ 0.50, 2.0 mL of a ferric salt solution (FeSO_4_, 5.20), and EDTA-Na_2_ (7.50). The standards of IAA and GA3 were purchased from J&K Scientific Company, Ltd., Beijing, China. High-performance liquid chromatography (HPLC)-grade methanol was purchased from Sigma-Aldrich. The cow manure compost and rapeseed meal for second solid-state fermentation were purchased from Nanyang Jupeng Biotechnology Co., Ltd., Nanyang, China. The soil samples S1 (watermelon had been planted for two consecutive years) and S2 (watermelon was never planted) were collected at a farmland (32°02′ N 118°47′ E) from the surface layer of 0–10 cm in Nanjing city, Jiangsu province, China, air dried, mixed, and passed through a 2 mm sieve. The soil properties are shown in Table S1.

### 2.2. The Isolation and Identification of Antagonistic Bacillus Strains against Fon

The potential antagonists of *Bacillus* against Fon were isolated from watermelon rhizosphere soils at the locations (32°52′ N 117°34′ E) in Chuzhou city, Anhui province, China. The soil adhered to roots gathered by gently shaking the rhizosphere soil samples. Five grams were ground to homogenate, suspended in sterile PBS buffer (Qingdao Hope Bio-Technology Co., Ltd., Qingdao, China), and shaken for 1 h. The mixture was diluted to the appropriate concentration, spread on LB (Difco) agar and incubated at 30 °C for 4 d, subsequently spraying the conidia of Fon to cover the surface of colonies, cultured in a 28 °C incubator for 48 h. The strains that could produce the bacteriostatic circle were selected and further purified by several streakings, then preserved at −80 °C in LB broth (Difco) supplemented with 15% (v/v) glycerol. One strain possessing the highest antagonistic activity, named XG-1, was isolated and identified according to its biochemical properties [27], combined with phylogenetic analysis [28].

### 2.3. The Antagonistic Effect against Mycelial Growth and Conidia Germination of Fon

After the isolation and identification of XG-1, we investigated the antagonistic effects against mycelial growth and conidia germination of Fon by plate confrontation experiments and microscopic examination. A 7-mm agar cut with the Fon was transferred onto the center of a fresh Potato Dextrose Agar (PDA) plate, and we inoculated the strain XG-1 in a straight line with the cut piece (2.5 cm distance), incubated at 25 °C for 5 d, and then we observed the bacteriostatic effect against mycelial growth. 

The conidia suspension of Fon was obtained as follows: a 1000 mL flask with 400 mL PDB medium was inoculated with a 7 mm agar cut covered with Fon, cultured on a rotary shaker at 200 rpm at 25 °C for 5 d to produce conidia. We filtered the mixture to remove the mycelium by gauze, centrifuged at 7000 rpm for 10 min, and dissolved in PBS buffer described above.

Strain XG-1 was grown in LB medium for 24 h at 30 °C on a rotary shaker (Shanghai Hesheng Instructment&Equipment Co., Ltd., Shanghai, China) (200 rpm), centrifuged at 12,000 rpm for 5 min to make the cells form precipitation. The supernatant was collected as an extracellular secretion of XG-1, mixed with a conidial suspension of Fon, and then transferred onto a hydrophobic membrane overnight at 25 °C. The conidia germination rate was determined by microscopic examination: under a four-fold microscope, we observed the germination of 100 random conidia in each visual field, and the germination rate was calculated by the average germination rate of 10 visual fields.

### 2.4. The Growth Promotion and Investigation of Promotion Substances in XG-1

The growth promoting of the bacterial strains has an important impact on the evaluation of the application value, including the field of bio-organic fertilizer, which can give it the ability of direct growth promotion; therefore, we identified the growth promotion and growth-related substances in XG-1. Cells of strain XG-1 were cultured in LB medium overnight, centrifuged at 5000 rpm, and resuspended with sterile water. The density was adjusted to 10^8^ colony-forming units (CFU)/mL for further experiments. The surfaces of the watermelon seeds (Meidu) were disinfected with 8% sodium hypochlorite for 3 min, washed with sterile water, transferred to sterile filter paper, and germinated for 2 d at 25 °C. Then, the germinated seeds were placed in Hoagland culture medium and incubated in a growth chamber with a 14 h light (28 °C) and 10 h dark (22 °C) photoperiod for 3 d, and transplanted to the pots containing 450 g soil (S2). Three treatments were set: CK, the watermelon seedlings were treated with 5 mL of sterile water; TR1, the seedlings were root-irrigated with 5 mL of XG-1 suspension; TR2, seedlings were root-irrigated with 10 mL XG-1 suspension. Ten replicates were performed. Three random seedlings were chosen after 50 d in the growth chamber. The seedling height, root lengths, and fresh weights of seedlings and roots were measured.

As for the identification of promoters, the phytase activity of XG-1 was measured as described by Shao et al. [29], and one unit (U) of phytase activity was defined as 1 μmol of phosphate per minute liberated under the assay condition. An inoculum (1%, v/v) of XG-1 suspension was added into 100 mL of Landy medium for the extraction of IAA or nutrient broth medium (NB) for GA3, incubated at 30 °C for 120 h, and a sample (4 mL) was collected every 24 h. The quantitative analysis of IAA and GA3 concentration is described in brief: the suspension was centrifuged at 8000 rpm, at 4 °C. Then we transferred the supernatant to another sterile Erlenmeyer flask, acidified the pH to 2.0–3.0 with HCl, and extracted with an equal volume of ethyl acetate three times. The sample was merged and centrifuged at 8000 rpm, 4 °C for 10 min, concentrated through rotary evaporation, dissolved in 0.5 mL methanol, filtered with a 0.22 μm membrane, and quantified by HPLC through the comparisons with values in the calibration curve.

HPLC was performed using an ultraviolet (UV) detector (Shimadzu, SPD-20A, Japan) at 220 nm for IAA, and 280 nm for GA3 with a mobile phase consisting of methanol/0.1% acetic acid (60:40, v/v) at a flow rate of 0.5 mL/min. The solutions (10 μL) were injected into the HPLC system for detection.

### 2.5. The Investigation of Solid State Fermentation Conditions of XG-1

After study of the antagonistic characteristics and growth-promoting effect of strain XG-1, the bio-organic fertilizer was prepared. Before the second solid-state fermentation experiment, it was necessary to determine the inoculation amount of the strain XG-1 and the additional amount of rapeseed meal, which can improve the resource utilization rate and adjust the C/N ratio. The system for determination of the inoculation amount consisted of 15 g cow manure compost and 15 mL sterile water, mixed evenly and put into a 100 mL triangular flask, inoculating the XG-1 suspension to the final concentrations of 0.5, 1.0, 1.5, 2.0, 2.5, and 3.0 × 10^7^ CFU/g, at 30 °C, 200 rpm for 5 d. One milliliter of the mixture was diluted to the appropriate concentration and coated onto an LB plate. Then, we recorded the number of colonies that emitted green fluorescence to determine the optimal amount of XG-1. Five repeats were performed.

The system for the addition of rapeseed meal consisted of 15 g cow manure compost (0%, 4%, 8%, 12%, 15%, and 18% of rapeseed meal already added, w/w) and 15 mL sterile water, mixed evenly and put it into a 100 mL triangular flask. The XG-1 suspension was inoculated to the final concentration of 1 × 10^7^ CFU/g, at 30 °C, 200 rpm for 5 d. The density of XG-1 was performed as described above. Five repeats were performed.

The matrix for the solid-state fermentation of XG-1 was composed of cow manure compost and rapeseed meal at the ratio of 85:15 (w/w), sterilized for 30 min at 121 °C and performed in a 2000 mL beaker. The water content of the matrix (45%, 50%, and 55%), fermentation temperature (25 °C, 30 °C, and 37 °C), and turning number (once, twice, and three times a day) were set, respectively, in three levels. The orthogonal experimental design (L9, 3^3^) was used for the determination of the optimal fermentation conditions (Table 1). The strain XG-1 was mixed into the sterile matrix to a final concentration of 1 × 10^7^ CFU/g, and incubated for 7 d. The density of XG-1 was performed as described above. Five repeats were performed.

### 2.6. Pot Experiments Analysis for Preventing the Watermelon Continuous Cropping Barrier

Pot experiments were performed in a greenhouse located in Nanyang, Henan province, China for 50 d with 32–35 °C day temperature, 20–23 °C night temperature, and the relative humidity at 60–80%. Fon could not be detected in the test soil S1 and S2. In order to simulate a more serious continuous cropping soil environment, Fon was inoculated to S1 samples at a final concentration of 10^5^ conidia/g soil using the conidia described above.

The watermelon seedlings were prepared as described in 2.4, and six treatments were designed: (1) soil S1, (2) soil S2, (3) soil S1 applied with novel bio-organic fertilizer, (4) soil S2 applied with novel bio-organic fertilizer, (5) soil S1 applied with cow manure compost, (6) soil S2 applied with cow manure compost. The novel bio-organic fertilizer or cow manure compost was mixed with soil at the ratio of 1:50. Three seedlings were planted in one pot containing 2.5 kg soil with the six treatments described. Each treatment was repeated 10 times. 

The disease symptoms of watermelon were observed and recorded at 25 d and 50 d, which were divided into five levels [21]: 0, no leaves were wilted; 1, <25.0% of leaves were wilted; 2, 25.0% to 50.0% of leaves were wilted; 3, 50.0% to 75.0% of leaves were wilted; 4, >75.0% of leaves were wilted. The disease index for each treatment was calculated according to the following formula: disease index = [Σ (rating × number of plants rated) × (total number of plants × highest rating)^−1^] × 100. At 50 d, the soil samples were collected as described in a previous study [30] and the density of XG-1 in rhizosphere and bulk soil was quantified. Fon was detected by quantitative polymerase chain reaction (qPCR) using the specific primers Fn-1 (5′-TACCACTTGTTGCCTCGGC-3′) and Fn-2 (5′-TTGAGGAACGCGAATTAAC-3′) [31]. The pH, organic matter, total N, total P, and total K of S1 rhizosphere soil applied with novel bio-organic fertilizer; then the watermelon growth under six treatments was determined. 

The relationship between the bacterial strains in bio-organic fertilizer and watermelon roots directly affects the survival of functional bacteria. Therefore, it is necessary to study the effect of the watermelon root exudates on the growth of strain XG-1 and the chemotactic response of strain XG-1 towards the root exudates and root colonization. The root exudates of watermelon planted in soil S1 applied with novel bio-organic fertilizer were collected to investigate the effect on the growth of XG-1 [32]. We inoculated a XG-1 bacterial suspension to 100 mL MSM medium to a final concentration of 10^7^ CFU/mL with the addition of 2 mL watermelon root exudates, cultured at 30 °C, 180 rpm for 120 h. Each sample was collected every 24 h for OD_600_ determination. Root colonization of XG-1 was observed qualitatively using a confocal laser-scanning microscope (CLSM, LeicaTCSSP3, Leica Microsystems Trading Co., Ltd., Shanghai, China) [32]. A qualitative detection of the chemotactic response of XG-1 towards the root exudates was also performed with minor amendments [33]. The activated culture of S113 was re-suspended in 20 mL semi-solid chemotactic buffer with a final concentration of 10^8^ CFU/mL, and poured into a plastic cell culture dish with a diameter of 6 cm (5 mL for each culture dish). After coagulation, a drop of concentrated root exudates (10 μL) was added to the center of the culture dish. A chemotactic buffer served as a negative control. The accumulation of XG-1 in the drop was observed after cultivation for 2 h. 

### 2.7. Statistical Analysis

The software IBM^®^ SPSS Statistics version 20 (International Business Machines Corporation, Armonk, NY, America) was used for statistical analysis. A one-way analysis of variance (ANOVA) test and Duncan’s multiple-range test were used to compare the means of the data. Multiple mean comparisons were performed with a significant difference test at *p* < 0.05. The values stated in tables and figures are the means of three replicates ± standard deviations.

## 3. Results

### 3.1. Strain Isolation and Identification

**Figure 1:**
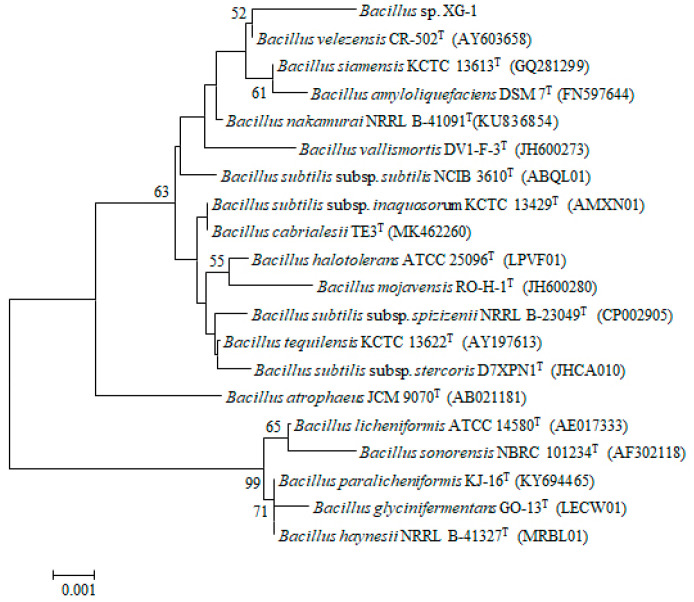
Neighbor-joining phylogenetic tree shows the taxonomic position of strain XG-1 and other closely related species of the genus *Lysobacter.*

Cells of the strain XG-1 are Gram-positive, rod-shaped and motile, and endospores are produced. Colonies are white, viscous, convex and circular with entire edges when cultured on LB agar at 30 °C for 2 d. Biofilm could be detected on the surface in LB liquid medium. Casein, aesculin, gelatin and starch were hydrolyzed, but negative results for the hydrolysis of Tween 80, H_2_S production. The strain XG-1 could utilize D-galactose, D-sorbitol, raffinose and melibiose for growth. The 16S rRNA gene sequence of XG-1 was compared with other sequences using the EzTaxon-e server (https://www.ezbiocloud.net/) [34]. Phylogenetic analysis based on the neighbor-joining statistical method showed that XG-1 clustered within the *Bacillus* species and formed a subclade closely related to *Bacillus velezensis* CR-502^T^ (99.79% similarity), *Bacillus siamensis* KCTC 13613^T^ (99.71% similarity) and *Bacillus amyloliquefaciens* DSM 7^T^ (99.50% similarity) (Figure 1). According to the phenotypic features, biochemical characteristics, and 16S rRNA gene phylogenetic analysis, the strain XG-1 was identified as a *Bacillus* sp., and chosen for further study.

### 3.2. The Inhibition of Mycelial Growth and Conidia Germination of Fon by XG-1

**Figure 2:**
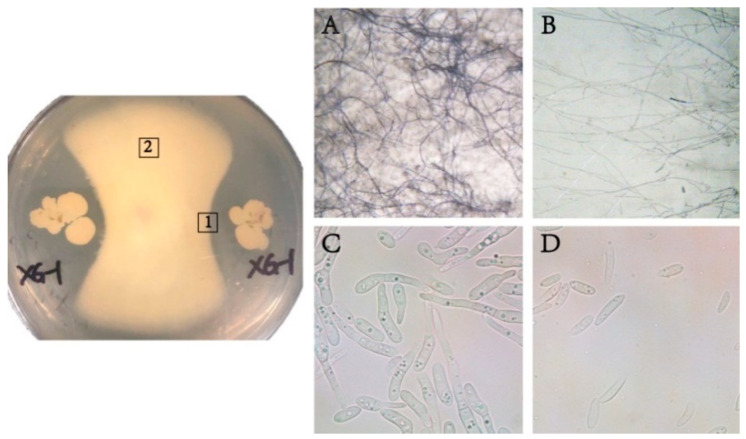
Antagonistic effect of strain XG-1 on conidia germination and mycelia growth of Fon. (**A**) and (**B**) showed the mycelia growth collected at places 2 and 1, respectively; (**C**): conidia germination treated with sterile water; (**D**): conidia germination treated with extracellular secretion of XG-1.

The strains of *Bacillus* used for biological control should have a strong inhibition effect on the pathogens of soil borne diseases; thus, the antagonistic characteristics of XG-1 were studied, which demonstrated a high level of antifungal activity against Fon. The inhibition of XG-1 on the mycelia growth of Fon was shown in Figure 2, forming an independent region to block the growth of mycelia (Figure 2A,B). The conidia germination of Fon could also be significantly inhibited (Figure 2C,D), with an inhibition ratio of 85.3% compared to the control without the treatment of XG-1.

### 3.3. The Investigation of Growth-Promotion Substances in XG-1

**Figure 3:**
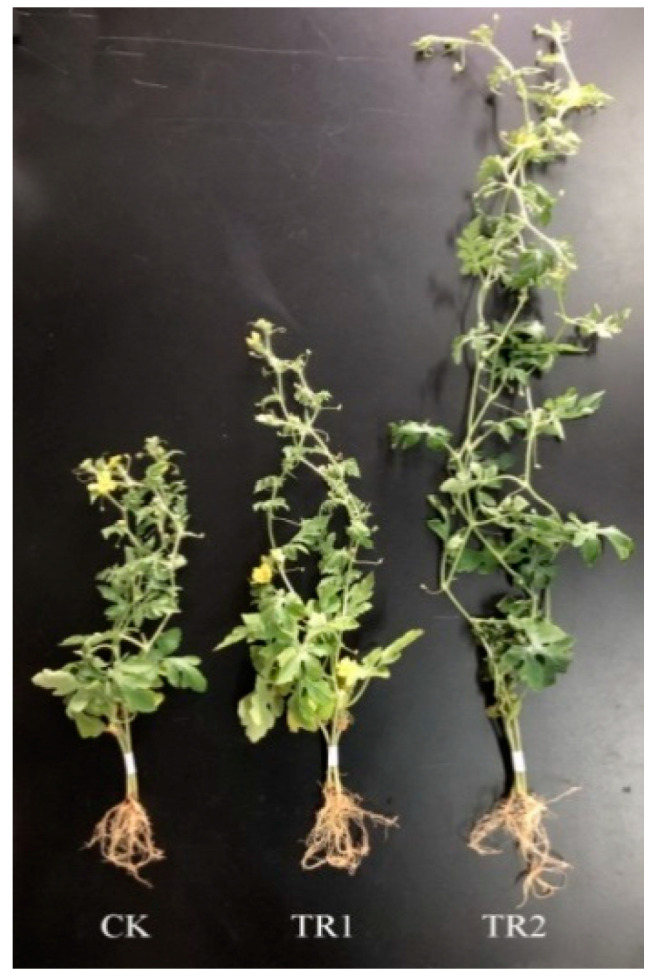
The promotion of watermelon by irrigation of XG-1.

The growth-promoting effect of XG-1 was assessed and shown in Table S2 and Figure 3, the growth indicators, including the seedling height, root length, seedling fresh weight and root fresh weight of watermelon (CK), were 35.2 ± 3.3 cm, 13.5 ± 2.1 cm, 14.28 ± 0.58 g, and 0.86 ± 0.12 g, respectively, cultivated for 50 d in a growth chamber. When applied with 5 mL of XG-1 suspension (TR1), the promotion effect was detected with an increase of 25.8%, 31.9%, 47.7%, and 27.9% in the seedling height, root length, seedling fresh weight, and root fresh weight, respectively. When 10 mL of the suspension was applied (TR2), the increase reached 99.4%, 72.6%, 121.1%, and 52.3%, which means the growth of watermelon was directly related to the strain XG-1, likely due to the secretion of growth-promoting substances or enzymes, regulating the growth of watermelon. 

**Figure 4:**
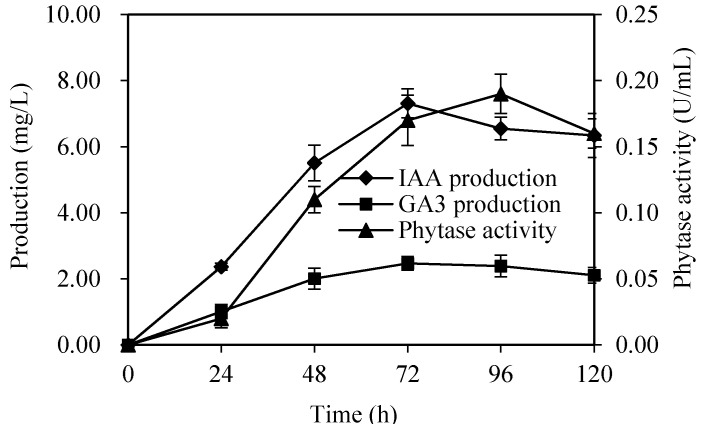
The phytase, indole-3-acetic acid (IAA) and gibberellic acid (GA3) production curve.

Subsequently, the potential growth promotion substances and enzymes were identified. IAA and GA3 are known as plant hormones, and the phytase could improve the availability of phosphorus by destroying the strong affinity of phytic acid and releasing phosphate residues. In this study, the phytase, IAA and GA3 production curves of XG-1 indicated that there existed at least three substances responsible for watermelon growth. The phytase was produced during the 120 h cultivation and the activity increased to the maximum at 96 h (0.19 U/mL), and slowly declined at 120 h (Figure 4). The IAA (Figure S1A) and GA3 (Figure S1B) production were characterized by HPLC and quantified as described above. The highest concentration of both IAA and GA3 appeared at 72 h, 7.31 mg/L for IAA and 2.47 mg/L for GA3 (Figure 4). These mainly accumulated in the stable period of the cell culture.

### 3.4. Preparation of Bio-Organic Fertilizer

The strain XG-1 can promote the growth of watermelon and strongly antagonize Fon, showing the potential to prevent the watermelon continuous cropping barrier. In order to prepare a novel bio-organic fertilizer based on the solid substrate of cow manure compost, the inoculation amount of XG-1 and the addition amount of rapeseed meal should be determined first, to achieve the goal of economic saving. As shown in Figure S2A, the optimal inoculation amount of XG-1 was 1.0 × 10^7^ CFU/g. After incubation at 30 °C for 5 d, the density reached nearly 2.5 × 10^7^ CFU/g, and no significant differences were found when exceeding this concentration. The optimal ratio of rapeseed meal added into the cow manure compost was 15% (Figure S2B), which could significantly increase the number of XG-1 found under the same conditions when compared with treatments without rapeseed meal.

Then, an orthogonal experiment (L9, 3^3^) containing water content of the matrix, fermentation temperature and turning number was designed for the determination of optimal conditions, and the results are shown in Table 2. The optimal level of each factor was A2 (fermentation temperature: 30 °C), B2 (water content: 55%), and C3 (twice one day for turning), respectively. Under this condition, the number of XG-1 was 4.62 × 10^8^ CFU/g, which was higher than the other eight combinations. Therefore, the optimal solid-state fermentation plan of strain XG-1 was A2B2C3, and the subsequent bio-organic fertilizer used for the prevention of watermelon continuous cropping barrier was chosen according to this program. The influence of experimental factors on the results analyzed by the value of range *R_j_* showed that the fermentation temperature had the greatest influence on the solid-state fermentation, followed by the turning number and the initial water content (*R_A_* > *R_C_* > *R_B_*).

### 3.5. Pot Analysis on the Prevention for the Watermelon Continuous Cropping Barrier by Bio-Organic Fertilizer

**Figure 5:**
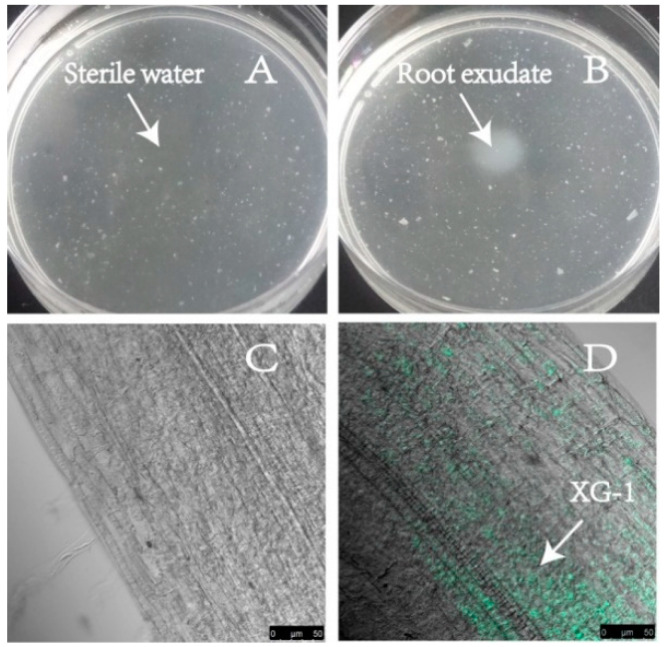
Chemotactic response towards root exudates and colonization on root surface by XG-1. (**A**): Drop with sterile water in the center; (**B**): Drop with root exudates in the center; (**C**): Watermelon root treated without bio-organic fertilizer; (**D**): Watermelon root treated with bio-organic fertilizer.

In order to test the control effect of bio-organic fertilizer based on cow manure compost fermented by XG-1, a pot experiment was set. The strain XG-1 in bio-organic fertilizer dispersed in test soil and moved to the watermelon rhizosphere depending on chemotactic response towards root exudates, which is regarded as a prerequisite for root colonization. The results of chemotactic response and colonization are shown in Figure 5, XG-1 exhibited positive chemotactic behavior to the watermelon root exudates, forming a high concentration of biospheres by XG-1 (Figure 5A,B), possibly due to the growth-promoting substances in the root exudates (Figure S3). Root colonization is one of the most important factors for functional strains to maintain competitiveness in the rhizosphere environment. As shown in Figure 5C,D, the strain XG-1 showed a strong colonization ability in the watermelon root system and gathered there. Thus, we speculated that the strain XG-1 in solid substrate, when added to the soil, migrated to the watermelon roots through chemotaxis and colonized, then utilized root exudates for growth, improved the biomass, and enhanced its viability in soil. The density of XG-1 in the rhizosphere soil was significantly higher than that in bulk soil, reaching 9.79 × 10^5^ CFU/g and 9.91 × 10^5^ CFU/g in the rhizosphere soil, respectively, for S1 + BOF and S2 + BOF. This resulted in the decrease of Fon in S1 + BOF (1.29 × 10^3^ copies/g) by more than one order of magnitude compared with the treatment without bio-organic fertilizer (S1, 1.15 × 10^5^ copies/g) or cow manure compost without secondary fermentation by XG-1 (S1 + CMC, 7.59 × 10^4^ copies/g) as revealed by qPCR (Table 3). The abundance trend of XG-1 and Fon in the bulk soil was consistent with that in the rhizosphere soil. The control efficiency of *Fusarium* wilt occurred in continuous cropping soil with bio-organic fertilizer (S1 + BOF) and reached 75.5% and 73.7% at 25 d, 86.4% and 86.1% at 50 d, compared with that in continuous cropping soil (S1) and continuous cropping soil treated with cow mature compost (S1 + CMC), respectively.

On the other hand, bio-organic fertilizer could also ameliorate the rhizosphere soil environment. Using the continuous cropping soil (S1) as the tested soil, the application of bio-organic fertilizer (S1 + BOF) enriched soil nutrients, including the organic matter (13.2%), total nitrogen (13.9%), total phosphorus (20.5%) and total potassium (3.77%) compared with S1, and also adjusted the soil pH from 6.69 to 7.01. Based on the results above, watermelon thrived under the action of bio-organic fertilizer, in both soil S1 and S2. For treatments related to S1, the increase rates of S1 + BOS in the seedling height, root length, fresh weight of seedling and root were 78.8%, 72.2%, 84.6%, and 96.4% (compared with S1) and 43.5%, 53.5%, 53.1%, and 64.2% (compared with S1 + CMC). Due to the growth-promoting effect of the strain XG-1, the four test indicators of watermelon in S1 + BOF were higher than those in S2, although there were no significant differences, showing that the bio-organic fertilizer could effectively control the continuous cropping barrier and ensure the growth of watermelon (Table 4). The cow manure compost without secondary fermentation by XG-1 (S1 + CMC) was not effective in preventing and controlling watermelon continuous cropping barrier, regardless of the disease incidence or watermelon growth.

## 4. Discussion

There are many situations that result in the continuous cropping barrier. The two abnormal changes in soil micro-ecosystem occur due to continuous cropping: abnormal microbial flora and chemical substances in soil. Among them, plant autotoxicity, deterioration of the soil physical and chemical properties, soil-borne diseases are the main factors [35]. The interaction between the factors aggravates the occurrence of continuous cropping barrier.

In some specific cases, plants will produce substances that inhibit their own growth, (called plant autotoxicity) [36], such as phenolic acids, a type of self-toxic substance, which widely exists in plant tissues, and is closely related to plant growth [37]. The phenolic acid secreted by the watermelon root system or formed in the process of residual plant decomposition, including ferulic acid, coumaric acid, cinnamic acid and vanillic acid, significantly inhibited the growth of watermelon seedlings and the germination of watermelon seeds [38,39], affected the soil pH. The watermelon growth experiments in this study showed that the strain XG-1 added to soil gathered through chemotaxis and colonized on roots, which was related to the growth of watermelon, likely due to the IAA, GA3, and phytase production after colonization. The highest concentrations of IAA and GA3 were 7.31 and 2.47 mg/L, respectively. The IAA production of XG-1 was lower than that of *Bacillus amyloliquefaciens* SQR9 induced by tryptophan (9.46 mg/L), but higher than that without tryptophan treatment (3.15 mg/L), which is more convenient in subsequent preparations of bio-organic fertilizer. SQR9 could also produce other promotion substances, such as acetoin, 2,3-butanediol, and phytase. The promotion rates of cucumbers in plant height and root length were 73.2% and 59.8%, respectively [29]. *Trichoderma helicum* significantly increased the watermelon height and root length by 46.0% and 30.6% [40]. Although the promotion substances detected in XG-1 were less than SQR9, the promoting rates of watermelon height and root length reached 99.4% and 72.6%, higher than the two strains above. The bio-organic fertilizer combined with different functional strains could also promote the growth of watermelon. Bio-organic fertilizer added with the suspension of *Paenibacillus polymyxa* and *Trichoderma harzianum* increased the watermelon’s fresh weight by 117.7% in comparison with control [23]. The bio-organic fertilizer fermented by the antagonistic microbe *Paenibacillus polymyxa* SQR-21 promoted the watermelon fresh weight from 48.82 g to 71.23 g (a 45.9% increase) [24]. *Bacillus amyloliquefaciens* JDF35 was used to prepare the bio-organic fertilizer, and the watermelon fresh weight was increased by less than 50.0% when treated with bio-organic fertilizer compared with the control [17], the increases for bio-organic fertilizer by *Paenibacillus jamilae* Cy5 and Fen-liquor Daqu were 63.0% and 9.7%, respectively [18,41]. In this study, the promoting rate of bio-organic fertilizer produced by XG-1 in watermelon fresh weight was 85.3%, which is not the highest among the three kinds of bio-organic fertilizer above, but also shows the potential application value. The strain XG-1 in the bio-organic fertilizer could secrete IAA, GA3 and phytase, which directly influenced the watermelon growth, but might also act as an important factor and cooperate with indigenous microorganisms to promote watermelon growth [42]. The root exudates of watermelon with good growth conversely improved the biomass of XG-1, forming a virtuous circle between watermelon and XG-1 to eliminate the inhibition of self-toxic substances.

Watermelon was susceptible to *Fusarium oxysporum* f. sp. *niveum* (Fon), which caused *Fusarium* wilt and could be aggravated by continuous cropping. More attention was paid to the biological control. In previous studies, different bacterial and fungal species, such as *Bacillus* sp., *Streptomycetes* sp., *Paenibacillus* sp., and *Trichoderma* sp., were used for prevention for Fon, mainly by antibiosis, competition, and the induction of systemic resistance. Antimicrobials produced by microorganisms were the primary mechanism of biological control. *Trichoderma harzianum* SQR-T037 inhibited Fon via producing volatile and non-volatile compounds [43]. Nearly 8.5% of the genome of *Bacillus amyloliquefaciens* FZB42 was related to the synthesis of antibiotics and siderophores [44]. Forty-two volatile organic compounds (VOCs) produced by *Paenibacillus polymyxa* WR-2 were identified including benzenes, alkyls, alcohols, ketones, aldehydes, acids, phenols, naphthalene and indole, with 5–100% antifungal activity [25]. The cell-free supernatant of *Bacillus* sp. WB inhibited the growth of Fon by affecting cellular morphology and disrupting the cell walls and membranes [15]. *Paenibacillus polymyxa* SQR-21 systemically affected watermelon root exudates to inhibit the conidial germination of Fon [45]. *Streptomyces goshikiensis* YCXU produced antifungal volatile compounds to disturb the growth of Fon up to 40% [5]. Lipopeptides (LPs) produced by *Bacillus* sp. were known to possess antifungal and antibacterial activities, which could cooperate or independently fight against pathogens. Within the strain of *Bacillus amyloliquefaciens* SQR9, one of the LPs, bacillomycin D, was proved to be accountable for the inhibition of *Fusarium oxysporum* [46]. In this study, the cell-free suspension of the strain XG-1 demonstrated a strong inhibitory effect on the growth of Fon conidial. Combined with previous studies, we speculate that the strain XG-1 can also produce LPs, but could not be identified through HPLC at the present. Although the antagonistic substances have not been identified, the bio-organic fertilizer produced by the strain XG-1 possessed a control efficiency of 86.4% for watermelon *Fusarium* wilt in pot experiments performed in a greenhouse, higher than those bio-organic fertilizers fermented by *Paenibacillus polymyxa* and *Trichoderma harzianum* (75.0%) [23], *Bacillus amyloliquefaciens* JDF35 (less than 60%) [17], Fen-liquor Daqu (66.7%) [18], and *Paenibacillus jamilae* Cy5 (75.0%) [41], slightly lower than that of *Paenibacillus polymyxa* SQR-21 (90.3%) [24].

The soil salinization and the change of soil properties are also considered to be important reasons for the continuous cropping barrier. Due to the selective absorption of chemical fertilizer by specific crops in continuous cropping, it is easy to develop a lack of certain elements, particularly trace elements. In protected cultivation, the salt accumulation caused by the excessive use of chemical fertilizer and lack of trace elements caused by ion antagonism will lead to growth barriers and reduce the quality and yield of crops [2]. In addition, the microbial population, soil enzyme activity and nutrient composition of continuous cropping soil deteriorate, becoming unsuitable for plant growth [47]. The application of bio-organic fertilizers has been proven to be efficient for supplying plant nutrients and improving soil fertility [48]. However, using the species of *Bacillus* as functional strains, the solid substrates of bio-organic fertilizer were primarily chicken manure compost, Chinese medicinal herb residue compost, amino acid organic fertilizer, and pig manure compost. The solid matrix fermented by *Bacillus amyloliquefaciens* SQR9 for the maximum amount of biomass consisted of 7.61% (w/w, DW, the same below) rapeseed meal, 8.85% expanded feather meal, 6.47% dewatered blue algal sludge, and 77.07% chicken compost, reaching 3.24 × 10^8^ CFU/g [20]. As for *Bacillus subtilis* SQR9, the optimal proportion of fermentation matrix leading to the maximum number of strains (1.97 × 10^10^ CFU/g) consisted of Chinese medicinal herb residue compost, cattle manure compost, pig manure compost and amino acid organic fertilizer (40:6:0:8, w/w/w/w) [19]. The bio-organic fertilizer based on amino acid fertilizer and pig manure composts (2:3, w/w) was produced by fermentation using *Bacillus amyloliquefaciens* W19 with a final concentration of 10^9^ CFU/g [7]. Cow manure shares the common characteristics of high water content, poor aeration, slow decomposition, low content of nutrients and organic matter, and has strict requirements for the fermentation strains. Zhao et al. demonstrated that the bio-organic fertilizer of cow and chicken manure compost (1:2, w/w) fermented by *Bacillus amyloliquefaciens* JDF35 and the bio-organic fertilizer of cow plus chicken manure compost (1:1, w/w) fermented by Fen-liquor Daqu altered the composition of the rhizosphere microbial community to suppress Fon [17,18]. The number of strain XG-1 in the bio-organic fertilizer consisting of cow manure compost and rapeseed meal obtained under optimal conditions could reach 4.62 × 10^8^ CFU/g, which was slightly lower than the number of strains obtained by fermentation with the other composts described above, but still demonstrated that XG-1 had a strong utilization and decomposition ability for the residual nutrients in cow manure compost and possessed higher control efficiency of Fon and growth promotion ability compared with the bio-organic fertilizer of cow plus chicken manure compost as described above. It is worth mentioning that the number of XG-1 also reached 4.75 × 10^8^ CFU/g using the purchased cow manure compost directly for secondary fermentation; however, the growth of watermelon was strongly inhibited when applied for pot experiments (data not shown). For this study, the cow manure compost was sterilized first and then fermented, which demonstrated a significant prevention for continuous cropping barrier, likely due to the high water content and incomplete maturity, which is suitable for the growth of various pathogenic fungi and bacteria, or due to remaining toxic substances, seriously affecting the quality of bio-organic fertilizer [49].

## 5. Conclusions

A bio-organic fertilizer consisting of cow manure compost and rapeseed meal, fermented by *Bacillus* sp. XG-1 (which was isolated from the watermelon rhizosphere soil) was found to eliminate the watermelon continuous cropping barrier by controlling *Fusarium* wilt, regulating the soil properties and enhancing the watermelon growth, showing potential application value in the watermelon production. However, more attention should be paid to the changes in the rhizosphere soil community, including the bacterial and fungal structure. The antagonistic ability against pathogens of other soil-borne diseases deserves to be tested for further study and application.

## Figures and Tables

**Table 1:** Factors and levels of the orthogonal test.

Level	Factor		
	A: Temperature (°C)	B: Water Content (%)	C: Number of Turning (/d)
1	25	45	0
2	30	50	1
3	37	55	2

**Table 2:** Tab of the orthogonal test.

Project	Factor			Y_i_
	A	B	C	
1	1	1	1	1.99
2	1	2	2	2.91
3	1	3	3	2.55
4	2	1	2	4.18
5	2	2	3	4.62
6	2	3	1	3.24
7	3	1	3	3.99
8	3	2	1	3.9
9	3	3	2	3.78
*k_j_*_1_	7.45	10.16	9.13	Y_i_
*k_j_*_2_	12.04	11.43	10.87	
*k_j_*_3_	11.67	9.57	11.16	
*K_j_*_1_	2.48	3.39	3.04	
*K_j_*_2_	4.01	3.81	3.62	
*K_j_*_3_	3.89	3.19	3.72	
*R_j_*	1.80	0.74	1.01	
Optimal level	A2	B2	C2	
Optimal project	A2B2C3			

Note: A: temperature. B: water content. C: number of turning. *k_ji_* is the sum of the data of corresponding level i in column j. *K_ji_* = *k_ji_*/3; *R_j_* = max (*K_j_*_1_, *K_j_*_2_, *K_j_*_3_) − min (*K_j_*_1_, *K_j_*_2_, *K_j_*_3_). Yi: the total number of XG-1 (× 10^8^ CFU/g)

**Table 3:** Disease incidences of watermelon and quantities of XG-1 and Fon in rhizosphere and bulk soil.

Treatment	Disease Incidence (%)		Strain XG-1 (50 d)		Fon (50 d)	
	25 d	50 d	Rhizosphere Soil (10^5^ CFU/g)	Bulk Soil (10^5^ CFU/g)	Rhizosphere Soil (Log_10_ copies g^−1^ soil)	Bulk Soil (Log_10_ copies g^−1^ soil)
S1	37.5 ± 3.1 b	79.2 ± 4.9 b	-	-	5.06 ± 0.22 b	4.97 ± 0.24 b
S2	-	-	-	-	-	-
S1 + BOF	9.2 ± 2.1 a	10.8 ± 1.5 a	9.79 ± 0.22 a	4.97 ± 0.15 a	3.11 ± 0.12 a	3.93 ± 0.21 a
S2 + BOF	-	-	9.91 ± 0.18 a	4.75 ± 0.11 a	-	-
S1 + CMC	35.0 ± 2.9 b	77.5 ± 5.1 b	-	-	4.88 ± 0.20 b	4.93 ± 0.17 b
S2 + CMC	-	-	-	-	-	-

Note: S1: soil S1, S2: soil S2, S1 + BOF: soil S1 applied with novel bio-organic fertilizer, S2 + BOF: soil S2 applied with novel bio-organic fertilizer, S1 + CMC: soil S1 applied with cow manure compost, S2 + CMC: soil S2 applied with cow manure compost. Data in the table are shown as the mean ± standard deviation. The different lowercase letters affixed to the data mean significant differences between the two treatments (*p <* 0.05).

**Table 4:** The growth of watermelon under different treatments.

Treatment	Seedling Height (cm)	Root Length (cm)	Fresh Weight of Seedling (g)	Fresh Weight of Root (g)
S1	24.0 ± 3.1 a	11.5 ± 3.0 a	8.31 ± 0.48 a	0.56 ± 0.11 a
S2	38.3 ± 5.7 c	14.3 ± 3.9 b	13.47 ± 0.39 b	0.81 ± 0.12 b
S1 + BOF	42.9 ± 4.3c d	19.8 ± 5.1 c	15.34 ± 0.62 bc	1.10 ± 0.27 c
S2 + BOF	44.1 ± 5.1 d	21.0 ± 3.3 c	17.11 ± 1.41 c	1.19 ± 0.37 c
S1 + CMC	29.9 ± 3.3 b	12.9 ± 2.2 ab	10.02 ± 1.06 a	0.67 ± 0.12 a
S2 + CMC	40.2 ± 3.2 c	18.9 ± 2.6 c	16.37 ± 1.21 c	1.05 ± 0.36 bc

Note: S1: soil S1, S2: soil S2, S1 + BOF: soil S1 applied with novel bio-organic fertilizer, S2 + BOF: soil S2 applied with novel bio-organic fertilizer, S1 + CMC: soil S1 applied with cow manure compost, S2 + CMC: soil S2 applied with cow manure compost. Data in the table are shown as the mean ± standard deviation. The different lowercase letters affixed to the data mean significant differences between the two treatments (*p* < 0.05) (*p* < 0.05).

## Supplementary Materials

The following are available online at https://www.mdpi.com/1660-4601/17/16/5714/s1, Table S1: Physical and chemical properties of test soil, Table S2: The growth of watermelon under different treatments, Figure S1: Detection of IAA (**A**) and GA3 (**B**) in XG-1, Figure S2: Determination of inoculation amount of XG-1 (**A**) and addition amount of rapeseed meal (**B**), Figure S3: The promotion of watermelon root exudates on the growth of XG-1.
