# Supplementary material for: The Prevention of Bio-Organic Fertilizer Fermented from Cow Manure Compost by Bacillus sp. XG-1 on Watermelon Continuous Cropping Barrier
Source: Int J Environ Res Public Health. 2020 Aug 7;17(16):5714. doi: 10.3390/ijerph17165714 (PMC7459639; doi:10.3390/ijerph17165714)
Supplement: Supplementary file 1 [file ijerph-17-05714-s001.pdf]

Table S1. Physical and chemical properties of test soil.

Soil	pH	Organic matter (g/kg)	Total N (g/kg)	Total P (g/kg)	Total K (g/kg)
S1	6.69	30.19	1.22	0.39	14.06
S2	6.97	35.77	1.57	0.53	15.18
S1+ BOF	7.01	34.18	1.39	0.47	14.59

Table S2. The growth of watermelon under different treatments.

Treatment	Seedling Height (cm)	Root Length (cm)	Fresh Weight of Seedling (g)	Fresh Weight of Root (g)
CK	35.2 ± 3.3 a	13.5 ± 2.1a	14.28 ± 0.58a	0.86 ± 0.12a
TR1	44.3 ± 4.1b	17.8 ± 3.6b	21.13 ± 0.39b	1.10 ± 0.16b
TR2	70.2 ± 4.9c	23.3 ± 4.1c	31.57 ± 0.42c	1.31 ± 0.21c

Note: CK, seedlings were treated with 10 mL sterile water; TR1, seedlings were root-irrigated with 5 mL XG-1 suspension; TR2, seedlings were root-irrigated with 10 mL XG-1 suspension. Data in the table are shown as the mean ± standard deviation. The lowercase letters affixed to the data mean significant differences ($p < 0.05$).

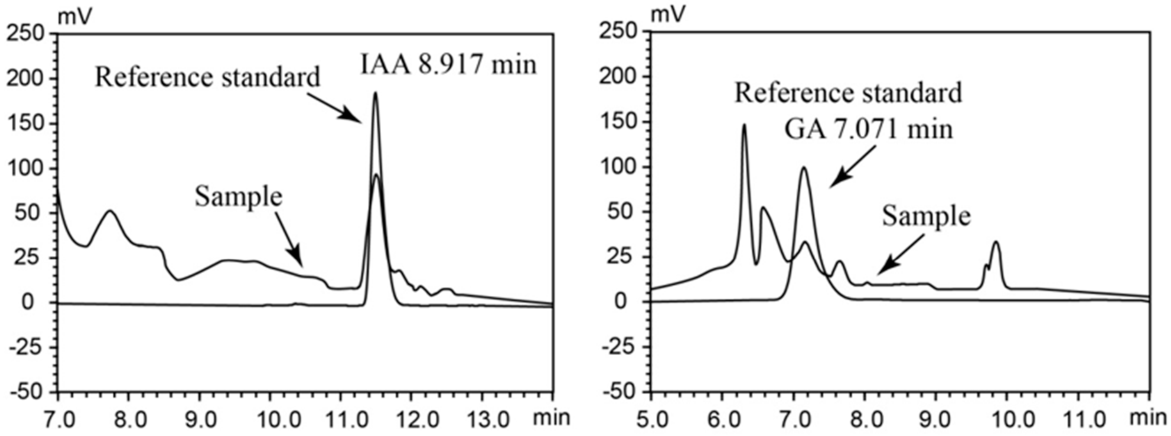

A B

Figure S1. Detection of IAA (**A**) and GA3 (**B**) in XG-1.

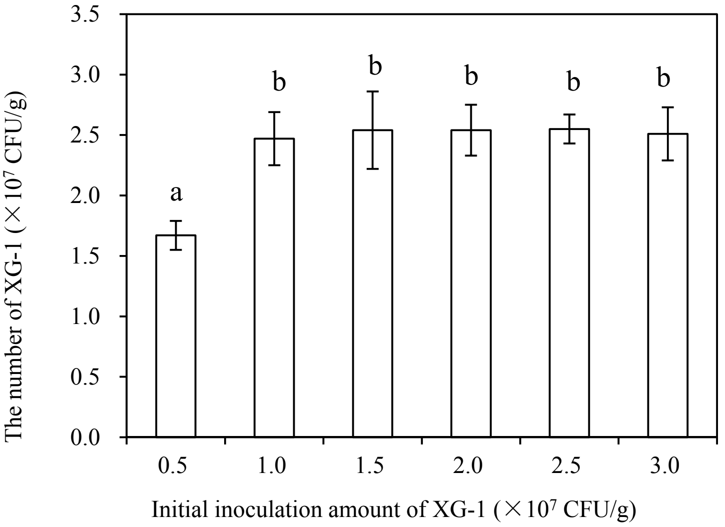

A

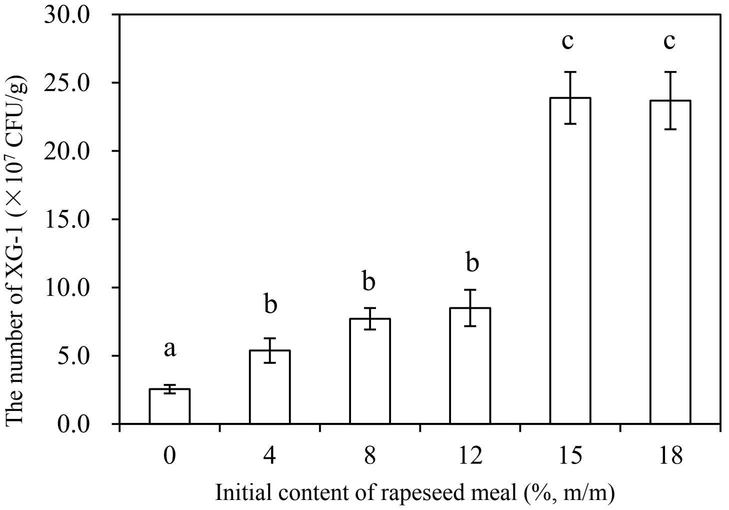

B

Figure S2. Determination of inoculation amount of XG-1 (**A**) and addition amount of rapeseed meal (**B**).

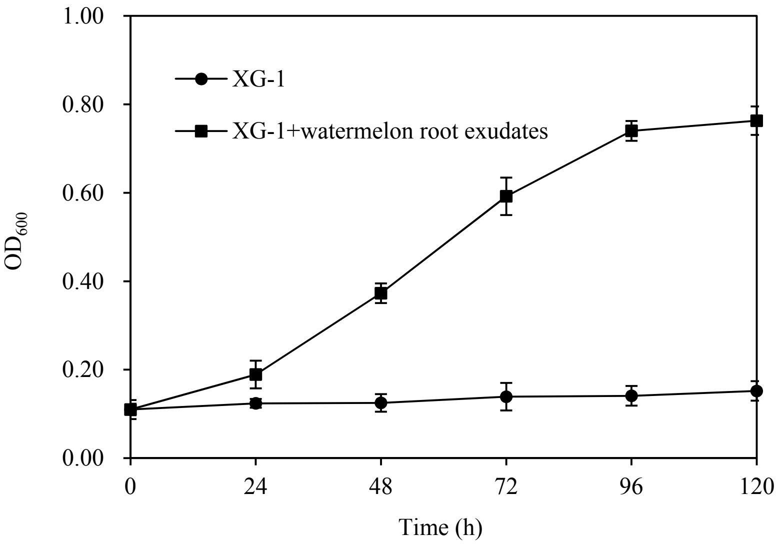

Figure S3. The promotion of watermelon root exudates on the growth of XG-1.
